# Treatment-Resistant Hepatitis C Viral Infection: A Case Report and Literature Review

**DOI:** 10.1155/2022/3556780

**Published:** 2022-03-11

**Authors:** Victoria Green, Marina Roytman

**Affiliations:** ^1^Department of Internal Medicine, University of California, San Francisco, Fresno, CA, USA; ^2^Department of Gastroenterology and Hepatology, University of California, San Francisco, Fresno, CA, USA

## Abstract

Hepatitis C virus (HCV) is an ongoing global public health threat affecting millions worldwide. Increasing recognition of its impact and recent advances towards HCV prevention and cure have provided incentive for the World Health Organization to call for global elimination by 2030. The goal of therapy is to achieve a sustained virologic response (SVR-12), defined as undetectable HCV-RNA within 12 weeks after treatment completion. In 2011, approval was given for the first direct-acting antiviral agents (DAAs). More recently, in 2013, more effective DAAs, with pan-genomic properties, have been introduced, and these regimens boast increasing rates of SVR. The ultimate goal is that the history of HCV ends with the pan-genotypic efficacy of multiple, easy-to-use and tolerate, combination regimens. These regimens have already demonstrated the ability to cure previously challenging patient groups. However, limitations exist in the current portfolio of agents, with suboptimal outcomes for patients with HCV genotype 3. In addition to this, access to DAAs remains an obstacle for many patients. We present this case of a 61-year-old male with HCV genotype 3 who has had several treatment failures with standard HCV therapy who was eventually approved for compassionate use of a 16-week course of glecaprevir (GLE)/pibrentasvir (PIB), sofosbuvir (SOF), and ribavirin (RBV) which ultimately led to SVR-12.

## 1. Introduction

Hepatitis C virus (HCV) is an ongoing global public health threat affecting millions worldwide [1]. Chronic HCV infection is one of the leading causes of liver-related death and one of the primary reasons for liver transplantation [2]. HCV transmission is most commonly associated with direct exposure to blood, via blood transfusions, unsafe health-care-related injections, and intravenous drug use [3]. Increasing recognition of its impact and recent advances towards HCV prevention and cure have provided incentive for the World Health Organization (WHO) to call for global elimination as a public health threat by 2030 [4]. This virus exhibits high genetic diversity, and within the six known genotypes, genotype 3 represents 22–30% of all infection and is distinctive as it is associated with higher rates of steatosis, accelerated fibrosis, and increased oncogenesis [5].

The goal of therapy is to achieve sustained virologic response (SVR), defined as undetectable HCV-RNA within 12 weeks after treatment completion, at which point the patient is considered cured. In 2011, approval was given for the first direct-acting antiviral agents (DAAs), boceprevir, and telaprevir, for treatment of genotype 1, in combination with interferon and ribavirin. More recently, in 2013, more effective DAAs, with pan-genomic properties, have been introduced, and these regimens boast increasing rates of SVR [2]. However, currently approved novel DAAs have not demonstrated the uniform potency seen in genotype 1 disease for genotype 3 [6]. Due to the distinctive characteristics of genotype 3, especially in patients with comorbid conditions, treatment often requires longer duration with decreased response rates. [7].

The ultimate goal is that the history of HCV ends with the pan-genotypic efficacy of multiple, easy-to-use and tolerate, combination regimens. These regimens have already demonstrated the ability to cure previously challenging patient groups, including human immunodeficiency virus-HCV coinfection, decompensated cirrhosis, and post-liver transplantation. However, limitations exist in the current portfolio of agents, with suboptimal outcomes for genotype 3 [8]. In addition to this, access to DAAs remains an obstacle for many patients. We present this case of treatment-resistant HCV GT3 for its contribution to the literature.

## 2. Case Summary

A 61-year-old man with a past medical history of chronic HCV leading to compensated cirrhosis and a subsequent diagnosis of non-Hodgkin's lymphoma (NHL) presented to the liver clinic for further evaluation and management. He likely contracted HCV in Vietnam from unregulated tattooing. He denies prior intravenous drug use or blood transfusion. The patient consumed 7–14 drinks a week up until a month prior to the presentation when he was advised to stop. He has a significant 30-pack-year smoking history and he continues to smoke half-pack of cigarettes daily. His family history is negative for liver disease or liver malignancies.

During the initial clinic visit, his vital signs were stable and he weighed 74.8 kg with a calculated body mass index of 23.68 kg/m^2^. His physical examination was positive for massive hepatosplenomegaly, however negative for scleral icterus, jaundice, ascites, peripheral edema, or asterixis. Laboratory studies of significant included creatinine 0.8 mg/dl, sodium 136 mmol/L, total bilirubin 1.5 mg/dl, international normalized ratio (INR) 1.2, platelets 115 10^3^/*μ*L, aspartate transaminase 107 U/L, alanine transaminase 73 U/L, and alkaline phosphatase 53 U/L. At this time, a model for end-stage liver disease (MELD) of 10 and Child-Pugh A were documented (see Table 1 for evolution of lab results).

Imaging included an abdominal ultrasound and magnetic resonance imaging/magnetic resonance cholangiopancreatography with and without contrast, both performed in 2018 and a computed tomography of the abdomen with and without contrast in 2019, all of which were consistent with cirrhosis and did not reveal any evidence of obstruction or malignancy. Small amount of ascites was noted on the 2019 imaging. The patient reports that, before entering this healthcare system, he was previously treated for HCV twice. In both treatment attempts, the viral load was undetectable during treatment and re-emerged after the discontinuation of antiviral therapy prior to 12-week posttreatment mark. The HCV genotype was retested each time and had remained GT3 which makes reinfection less likely and this is also compounded by his lack of risk factors for re-infection at the time. In 2019, his HCV-RNA was initially 338,000/IU and he was treated with a combination of sofosbuvir (SOF) and velpatasvir (VEL) for a duration of 12 weeks without achieving SVR-12. Subsequently, a combination of sofosbuvir/velpatasvir/voxilaprevir (SOF/VEL/VOX) and ribavirin (RBV) for a duration of 12 weeks was attempted and again did not lead to SVR-12. The patient reports meticulous compliance with both rounds of treatment. In both treatment attempts, the viral load was undetectable during treatment and re-emerged after the discontinuation of antiviral therapy prior to 12 weeks posttreatment. He underwent NS5A resistance testing which documented S62A and Y93H mutations predictive of resistance to VEL and daclatasvir (DCV). A 16-week combination therapy of glecaprevir/pibrentasvir (GLE/PIB) with SOF and RBV led to SVR-12 (see discussion below for the rationale).

With respect to complications of advanced liver disease, he has never experienced jaundice, clinically significant ascites or spontaneous bacterial peritonitis, variceal hemorrhage, or hepatic encephalopathy. Screening upper endoscopy did not reveal varices. He was diagnosed with NHL in 2019, which was attributing to ongoing HCV viremia. He had completed rituximab, cyclophosphamide, doxorubicin, vincristine (oncovin), and prednisolone (R-CHOP) therapy prior to achieving SVR-12 and is currently free of cancer as per his latest positron emission tomography scan.

## 3. Discussion

Achieving SVR improves overall survival and reduces the risk of liver failure, fibrosis progression, liver transplantation, and the incidence of hepatocellular carcinoma (HCC) [9]. Genotype 3 HCV infection is the second most prevalent HCV infection. However, cure rates are lower in some patients with genotype 3 such as those with cirrhosis or prior treatment experience. This therapeutic challenge may be in part explained by its unique characteristics such as its association with insulin resistance and alterations in lipid metabolism [10]. This patient was initially treated with SOF and VEL, a combination which in the ASTRAL-3 study led to SVR12 in 88% of participants with baseline velpatasvir-specific resistance associated substitutions (RASs) compared with 97% in those without NS5a RASs [11]. The patient did not achieve SVR12 leading to concern about the presence of NS5A RASs. As a result of the lower SVR12 in treatment-experienced individuals with cirrhosis in the ASTRAL-3 study, the addition of RBV was recommended for those with both treatment experience and cirrhosis [12].

DAA regimens containing NS5A inhibitors are highly effective treatments for HCV infection, but they are not 100% successful. Of the major HCV antiviral drug classes, there is only compelling evidence for the impact of NS5A inhibitor RASs on treatment outcome. In the POLARIS-1 phase 3 study, SOF/VEL/VOX for 12 weeks was highly effective in obtaining SVR in patients previously treated with a DAA regimen containing an NS5A inhibitor [13]. This multiclass combination given for 12 weeks without RBV led to a cure in 96% of the HCV infected who had a prior NS5A treatment failure [13]. This combination of SOF/VEL/VOX plus RBV was the second regimen offered to our patient, but again, it did not result in SVR-12.

According to Wyles et al., when NS5A inhibitor-based treatment fails, NS5A RASs frequently emerge and the presence of cirrhosis and prior HCV treatment increases the clinical impact of NS5A RASs [14]. Following failed NS5A-based treatment, up to 90% of individuals have NS5A RASs and they persist in most individuals for several years [15, 16]. Fortunately, the impact of NS5A RAS is relative and can often be overcome by increasing the length of therapy or by adding RBV [17]. NS5A resistance testing is recommended at the time of failure of NS5A inhibitor-based treatment. The NS5A RAS of most clinical importance in HCV genotype 3 is Y93H, which confers a high level of resistance to DVR and VEL [18, 19]. In the case of virologic failure following treatment exposure to an NS5A inhibitor, the Y93H RAS emerges in the majority of individuals [14]. Our patient underwent NS5A resistance mutation testing which documented S62A and Y93H. VEL has an improved resistance profile compared with early generation NS5A inhibitors, although the Y93H variant in HCV genotype 3 infection still confers a high level of resistance to VEL [20]. Notably, data on the impact of baseline NS5A RASs on the outcome of NS5A inhibitor-containing therapy are limited.

This patient's treatment course was complicated by the development of diffuse large B-cell lymphoma (DLBCL), the most common type of NHL. The association between HCV and NHL is well documented and supported by epidemiological studies [21]. The exact pathogenetic mechanism is still unknown, but several mechanisms of HCV-related lymphomagenesis have been postulated [22]. The optimal management of HCV-related NHL is unclear; however, antiviral treatment may be satisfactory for low grade or asymptomatic cases, while immunotherapy is warranted in high grade cases [23]. The R-CHOP regimen is the gold standard for DLBCL as it has been shown to improve the clinical outcomes and prognosis [24]. Clinically significant flares of HCV during immunosuppressive therapy remain exceedingly uncommon, our patient did not experience it. He tolerated treatment well and was found to be free of cancer according to his latest positron emission tomography scan report.

Options for retreatment after failure of an NS5A inhibitor-based regimen are challenging. Newer generation NS5A inhibitors such as PIB retain activity against all NS5A RASs and therefore may retain activity despite resistance to current NS5A inhibitors [25]. Tailoring retreatment based on results of NS5A and NS3 resistance testing is recommended. As such, NS3 resistance testing may be an explorable avenue for this patient. The combination of GLE/PIB is the first pan-genotypic NS3/4A protease inhibitor-NS5A inhibitor combination to be approved that offers a potent ribavirin-free option [26]. A 2020 study by Toyoda et al. showed that the 12-week GLE/PIB regimen showed high virologic efficacy in patients with cirrhosis, experience of DAA, or HCV genotype 3 [27]. The MAGELLAN-3 trial showed that the addition of SOF and RBV led to SVR in 95% of patients [7, 14]. We felt that a combination of GLE/PIB with SOF plus RBV would offer our patient the best chance of cure based on his prior treatment experience and resistance analysis.

This combination therapy (GLE/PIB/SOF/RBV) was repeatedly denied by the patient's insurance due to the experimental nature of it and due to the patient having reached a lifetime limit of HCV treatment. Our concern for liver disease progression as manifested by the development of radiologically detectable ascites as well as concern to recurrence of NHL in the setting of ongoing viremia led to a last-ditch effort to obtain this combination through an appeal for compassionate use directly from pharmaceutical companies. The Food and Drug Administration (FDA) allows companies to provide their drugs to patients under certain circumstances such as having a serious disease, or whose life is immediately threatened by their disease or there is no comparable or satisfactory alternative therapy to diagnose, monitor, or treat the disease, and this is referred to as “compassionate use.” Gaining access to these drugs through a compassionate use request can be a long and challenging process. Most patients will respond to treatment with SOF/VEL according to the ASTRAL-3 study [4]. In the POLARIS-1 phase 3 study, 96% of patients who fail SOF/VEL will achieve SVR with SOF/VEL/VOX [5]. This patient failed to respond to both regimens potentially due to presence of Y93H, the most clinically important resistance associated substitution [6]. In addition to this, the development of NHL also contributed to his complicated course.

In November 2020, compassionate use with third round of treatment for HCV with a 16-week course of GLE/PIB 100–40 mg daily, SOF 400 mg daily, and weight-based RBV at 600 mg twice daily was approved. The patient commenced therapy in January 2021 and continued to undergo close monitoring of liver enzymes, liver function tests, renal function tests, complete blood count, and HCV RNA every 2 weeks. The patient tolerated his 16-week treatment well with the only side effect endorsed being insomnia. August 2021 marked 12 weeks after the completion of treatment at which point his HCV RNA remained undetectable and he was considered cured. Reemergence of the virus after SVR-12 is extremely uncommon. According to Lin et al., PPV and NPV of SVR-12 predicting SVR-24 were 99.9% and 100%, respectively [28]. Retesting of HCV-RNA is generally driven by an unexplained rise in liver enzymes or high-risk behaviors. Neither of these situations is the case for the patient described in this case report. However given the rarity of this case, HCV-RNA will be retested at the SVR-24 time point.

The road to SVR for our patient was a difficult one, with several treatment failures and insurance obstacles. We remain hopeful for newer advancements that may benefit patients in this and similar predicaments.

The emergence of DAAs has made treatment effective and safe; however, treatment is costly and often inaccessible. Combining drugs from different classes produced by different pharmaceutical companies creates added barriers. In particular, individuals with HCV genotype 3 infection in whom NS5A inhibitor-based treatment has failed remain a population for whom we should increase our efforts to understand the virology and pathogenesis of, aiming at enhanced and more potent genotype-targeted treatments.

## Figures and Tables

**Table 1:** The evolution of pertinent lab results: pretreatment, during treatment, and posttreatment.

Labs	Dates													
	Pretreatment							During treatment				Posttreatment		
	Apr-19	Aug-19	Dec-19	Jan-20	Jul-20	Sep-20	Jan-21	Feb-21	Mar-21	Apr-21	May-21	Jun-21	Aug-21	Sep-21
AST, U/L	107	47	72	87	108	94	93	109	81	49	44	70	49	51
ALT, U/L	73	27	44	51	64	55	48	66	50	36	31	43	28	30
ALP, U/L	53	53	57	78	81	80	112	99	83	77	83	84	96	108
Total bilirubin, mg/dL	1.5	1	1.0	1.1	1.9	1.5	1.2	1.2	2.3	2.6	2.8	1	1.3	1.3
PT/INR	1.2	1.2	NT	NT	NT	1.2	1.1	1.2	1.2	1.1	1.1	1.2	1.2	1.1
Platelets, 10^3^/*μ*L	115	197	67	54	78	100	106	108	103	107	117	105	92	107
Albumin, g/dL	3.6	4.2	3.5	3.3	3.4	3.6	3.3	3.6	3.6	3.5	3.6	3.8	3.6	3.5
Creatinine, mg/dL	0.8	1.4	0.7	0.6	0.9	0.8	0.8	1.0	0.9	0.8	0.8	0.9	1.2	0.9
eGFR, mL/min/1.73 m^2^	>90	62	52	>90	85	92	95	85	85	85	93	83	61	80
Sodium, mmol/L	136	137	136	135	135	134	134	135	136	135	138	135	136	136
HCV-RNA quant (IU)	338,000	NT	6,510,00	NT	1,550,000	NT	NT	NT	ND	ND	ND	ND	ND	ND

NT, not tested; ND, not detected.
